# Is socioeconomic position associated with risk of attempted suicide in rural Sri Lanka? A cross-sectional study of 165 000 individuals

**DOI:** 10.1136/bmjopen-2016-014006

**Published:** 2017-03-22

**Authors:** D W Knipe, D Gunnell, R Pieris, C Priyadarshana, M Weerasinghe, M Pearson, S Jayamanne, A H Dawson, F Mohamed, I Gawarammana, K Hawton, F Konradsen, M Eddleston, C Metcalfe

**Affiliations:** 1 School of Social and Community Medicine, University of Bristol, Bristol, UK; 2 Faculty of Medicine, South Asian Clinical Toxicology Research Collaboration (SACTRC), University of Peradeniya, Peradeniya, Sri Lanka; 3 Faculty of Medicine & Allied Sciences, Department of Community Medicine, Rajarata University of Sri Lanka, Anuradhapura, Sri Lanka; 4 Clinical Pharmacology Unit, University of Edinburgh, The Queen's Medical Research Institute, Edinburgh, UK; 5 Faculty of Medicine, University of Kelanyia, Kelanyia, Sri Lanka; 6 Central Clinical School, University of Sydney, Sydney, Australia; 7 Translational Australian Clinical Toxicology (TACT) Group, School of Medical Science, University of Sydney, Sydney, Australia; 8 Faculty of Medicine, Department of Medicine, University of Peradeniya, Peradeniya, Sri Lanka; 9 Department of Psychiatry, Centre for Suicide Research, University of Oxford, Oxford, UK; 10 Faculty of Health and Medical Sciences, Department of Public Health, University of Copenhagen, Copenhagen, Denmark

**Keywords:** MENTAL HEALTH, EPIDEMIOLOGY, PUBLIC HEALTH, SOCIAL MEDICINE

## Abstract

**Background:**

Lower socioeconomic position (SEP) is associated with an increased risk of suicidal behaviour in high-income countries, but this association is unclear in low-income and middle-income countries.

**Methods:**

We investigated the association of SEP with attempted suicide in a cross-sectional survey of 165 233 Sri Lankans. SEP data were collected at the household (assets, social standing (highest occupation of a household member), foreign employment and young (≤40 years) female-headed households) and individual level (education and occupation). Respondent-reported data on suicide attempts in the past year were recorded. Random-effects logistic regression models, accounting for clustering, were used to investigate the association of SEP with attempted suicide.

**Results:**

Households reported 398 attempted suicides in the preceding year (239 per 100 000). Fewer assets (OR 3.2, 95% CI 2.4 to 4.4) and having a daily wage labourer (ie, insecure/low-income job; OR 2.3, 95% CI 1.6 to 3.2) as the highest occupation increased the risk of an attempted suicide within households. At an individual level, daily wage labourers were at an increased risk of attempted suicide compared with farmers. The strongest associations were with low levels of education (OR 4.6, 95% CI 2.5 to 8.4), with a stronger association in men than women.

**Conclusions:**

We found that indicators of lower SEP are associated with increased risk of attempted suicide in rural Sri Lanka. Longitudinal studies with objective measures of suicide attempts are needed to confirm this association.

**Trial registration number:**

NCT01146496; Pre-results.

**Strengths and limitations of this study**

- This is the first large (n>165 000) representative study in a general population (no age/gender restrictions) sample investigating the associations of socioeconomic position (SEP) with attempted suicide in a South Asian country.
- This study did not concurrently adjust for several measures of SEP, and therefore was able to provide a better estimate than previous studies of the association of SEP with suicidal behaviour in this region.
- The analysis investigates for the first time whether SEP associations differ in men versus women in this region in a general population sample.
- The findings rely on respondent report for the measure of outcome, and may be subject to reverse causality due to the cross-sectional design.

## Introduction

Suicide is a major cause of premature mortality in low-income and middle-income countries (LMIC) in South and South-East Asia; 55% of the world's suicides occur in these countries.^1^ A better understanding of the risk factors for suicide and attempted suicide in these settings is essential for developing informed suicide prevention policies.

The epidemiology of suicide in LMIC differs from that in high-income countries (HIC). Unlike HIC, many LMIC have high suicide rates in young people, particularly women.^2^ ^3^ In high-income South-East Asian countries such as Japan and South Korea, the gender ratio for suicide is higher (male:female 2.7 and 2.3, respectively) than when compared with the two largest LMIC in Asia, India (1.6) and China (0.8).^1^ Nevertheless, gender ratios for suicide attempts are similar in HIC and low and middle income South-East Asian countries, with higher rates in women than men.^4^ ^5^ One explanation for the difference might be differences in the methods used in acts of self-harm. Pesticide poisoning is a frequent method of suicide in LMIC, and often used in acts of self-harm with relatively low suicidal intent.^6^ ^7^ In contrast, in South-East Asian HIC, like Hong Kong and Singapore, the most common means of suicide is jumping from a height,^8^ and the most common method of suicide attempts is by medicinal poisoning.^4^ Given the relatively high case fatality of methods (ie, pesticide poisoning) used in acts of self-harm in LMIC, the epidemiology of suicide may be more similar to that of self-harm/attempted suicide than it is in HIC.^9^ It has also been argued that there are important cultural differences in suicidal behaviour in Asia to that of western HIC, and therefore the associations of risk factors with suicidal behaviour may differ.^10^

Socioeconomic position (SEP) has a significant role in suicidal behaviour in HIC, with some studies reporting up to a fivefold greater risk in individuals with lower SEP.^11^ We define SEP to include both the social and economic factors that are important in determining a person's place in society (ie, how they perceive their own status and how they are recognised by others).^12^ SEP is strongly associated with material wealth and it may be this, rather than an individual's ‘place’ in society, that influences suicide risk*.* It has been previously argued that the association of risk factors with suicidal behaviour in Asia is different to that observed in more westernised HIC.^13^ A recent systematic review concluded that the nature of this relationship in LMIC in South and South-East Asia is unclear, and that compared with the rest of the world, relatively few studies have examined this association in Asian LMIC^14^ and those that have, had several limitations. Some studies compared the SEP characteristics of suicide deaths with other causes of death, but this underestimates the association with SEP as most other causes of premature mortality are likely to be strongly socially patterned.^3^ ^15^ Other studies have focused on a subset of the population (ie, just women or older/younger individuals) or adjusted for multiple measures of SEP in the same analysis;^14^ the latter approach is likely to reduce/underestimate the association between the measure of SEP and suicidal behaviour. The systematic review did not identify a single study from Sri Lanka (a country which had one of the highest suicide rates in the world in the mid-1990s) which investigated the association of suicidal behaviour with a conventional (ie, education/occupation) measure of SEP. In a recent systematic review of suicide and poverty in LMIC, the need for more country-specific studies investigating this association is highlighted.^16^

Studies from HIC indicate that there may be gender differences in the association of SEP with suicide/attempted suicide. Studies have also shown that associations of suicide and low-income level, education and employment/occupation status were stronger in men than women.^11^ ^17^ ^18^ Gender differences in the association of SEP with risk of suicide/attempted suicide in South Asian LMIC are unclear. It is important to understand whether associations differ between men and women as this may allow appropriate planning of public health interventions.

In this study, we used data collected as part of a large cluster randomised control trial in Sri Lanka^19^ to investigate: (1) the association of SEP with attempted suicide risk in the general population using SEP measures at the individual and household level; (2) gender and age differences in SEP-related risk of attempted suicide.

## Methods

### Sri Lanka

Sri Lanka is a lower middle-income country situated off the south-east coast of India, with a very high literacy rate (96%—Sri Lanka Census 2011) and a high life expectancy (75 years in 2014—World Bank Data). Its closest neighbour India has a literacy rate of 74% (India Census 2011) and life expectancy of 68 years (2014—World Bank Data). The main suicide prevention efforts in Sri Lanka have included the establishment of a Presidential Committee, legislative changes (primarily around availability of toxic pesticides) and improved clinical management of people who attempt suicide.^20^

### Participants

Data used in this analysis were collected as part of the baseline survey for a community-based cluster randomised controlled trial to evaluate the effectiveness of safe storage devices in reducing self-poisoning with pesticides in a rural area of the Anuradhapura district of Sri Lanka.^19^ ^21^ Data were collected between 31 December 2010 and 2 February 2013. All individuals living in the study area were eligible for inclusion. The details of the baseline survey have been previously described.^19^ ^21^ Briefly, we conducted a door-to-door household survey with a team of high school (A-level) graduates (maximum 25 data collectors). The interview was conducted as a face-to-face interview between the data collector and an adult (≥18 years) household member. For pragmatic reasons, the survey was conducted by interviewing any (and sometimes more than one) member of the household, based on their availability at the time the data collector visited. Verbal consent for the household to participate was taken from the over 18 years old responding to the survey. We recorded which household members were present at the time of the survey*.* Data collectors received regular refresher training, audits and feedback on data quality.^21^ The interview was conducted within the compound of the respondents' home in their local language (Sinhala/Tamil). If no household member was available for interview, we returned to these households (at different times of the day) a minimum of three times to limit bias.

For logistical purposes, the study area was split into 10 regions/bands. We only included data collected from bands 2–10, as the data in band 1 for our outcome (suicide attempt) and one measure of SEP (household construction) were collected using slightly different definitions.

### Interview data

The survey included data on characteristics of the household and each household member. We also recorded data on suicide attempts reported to have occurred in the past year by household members. Details of the questions asked (translated into English) can be found in the online supplementary material methods; survey data were recorded directly onto hand-held devices.^21^ To ensure the quality of the data collected, we carried out regular inter-rate reliability checks (3% of all households were resurveyed). We also carried out automated data record checks to highlight data entry errors, missing fields and data inconsistencies.

10.1136/bmjopen-2016-014006.supp1 supplementary data

### Measures of SEP

#### Household measures

Composite asset score—a composite asset score was derived by combining data on household construction and motorised vehicle ownership (see online supplementary material methods for details). In order to create a single asset score, we dichotomised vehicle ownership and household construction, and combined these into a total score. These categories allowed us to create an ordinal variable with three levels—low (no motorised vehicle AND poor quality household construction); middle (either a motorised vehicle OR moderate-quality/high-quality household construction) or high (motorised vehicle ownership AND moderate-quality/high-quality household construction) asset ownership.

Highest ranking occupation of a household member—the highest occupation of a household member was used as a measure of social standing of the whole household. The ranking of occupations was derived by community focus groups in the area (see online supplementary material methods). Table 1 gives the occupation categories according to the ranking developed. Individuals were classed as having a salaried occupation if they received a monthly salary from a company and were not government employees, overseas workers or in the security forces, as these were separate categories in this data set. Examples of salaried employees were shop workers, insurance salespersons and bank employees. Self-employed individuals were skilled people who worked for themselves with no employees. Examples of self-employed individuals were carpenters and builders. Daily wage labourers were primarily unskilled individuals who were engaged in menial tasks and received a daily wage; there are no contracts associated with this type of occupation (ie, no security) and they tend to receive a low wage. Domestic workers, agricultural workers and unskilled manual labourers were grouped into this category.

**Table 1:** Household-level and individual-level SEP of total sample and respondent-reported suicide attempts in the past year

	Total n	Suicide attempters n (%)
*Household measures*		
Asset score		
High	107 324	192 (0.2)
Middle	48 368	153 (0.3)
Low	9541	53 (0.6)
Highest occupation in household*		
Government worker/graduate foreign employed	20 025	27 (0.1)
Farmer	73 681	165 (0.2)
Security forces	15 912	39 (0.2)
Businessmen	6266	10 (0.2)
Self-employed	19 262	57 (0.3)
Non-graduate foreign employed	4882	12 (0.2)
Salaried employee	11 603	30 (0.3)
Daily wage labourer	8985	46 (0.5)
Unemployed/retired	3519	7 (0.2)
Other†	1098	5 (0.5)
Young female head of household (≤40 years)	2472	11 (0.4)
Household with non-graduate foreign employed	16 858	36 (0.2)
*Individual measures*		
Age group (years)		
10–25	50 533	199 (0.4)
26–40	52 171	135 (0.3)
41–55	36 968	53 (0.1)
56+	25 561	11 (0.04)
Individual occupation*		
Government worker/graduate foreign employed	6276	4 (0.1)
Farmer	28 669	56 (0.2)
Security forces	8456	7 (0.1)
Businessmen	3386	2 (0.1)
Self-employed	11 211	34 (0.3)
Non-graduate foreign employed	4431	3 (0.1)
Salaried employee	14 023	52 (0.4)
Daily wage labourer	9380	45 (0.5)
Unemployed/retired	18 171	63 (0.3)
House worker/other	35 440	90 (0.3)
Student	25 790	42 (0.2)
Individual education		
University/A-level	30 150	51 (0.2)
O-level	105 403	281 (0.3)
Primary	24 542	51 (0.2)
Not attended	5138	15 (0.3)

*Presented according to ranking.

†Made up of house workers, students and other occupations. There were no attempts in households with student or other as the highest occupation.

SEP, socioeconomic position.

Young female heads of household—each household has a head of household, dictated by gender and generational hierarchies. The reporting of a young female head of household by a household member is rare because of cultural norms. A female-headed household is more likely to be socially and materially disadvantaged;^22^ this is particularly the case for young and middle-aged female-headed households. The average age of a female head of household in our data set was 50 (SD 18) years. There is no agreed definition of the age cut-off for defining a young female head of household; we chose a cut-off of ≤40 years. We also investigated whether a different cut-point for defining young female heads of household (≤30 years (n=320) and ≤50 years (n=8234)) influenced the association with this measure.

Non-graduate foreign employed—migration to the Middle East for non-professional occupations is a common phenomenon in Sri Lanka. In the Anuradhapura district, 60% of temporary migration for employment was by women (Census 2011). Migration, particularly of women, is thought to lead to disruptions to the family and has been linked to suicide risk, both of the migrants themselves and those left behind.^23^ We used foreign migration of a non-graduate as an indicator of migration for non-skilled work.

#### Individual measures

The individual SEP measures were: (1) highest educational qualification; and (2) occupation. Educational qualifications were categorised into four groups: (1) not attended school; (2) primary school only; (3) completed ordinary level (O-level)—examinations are taken around the age of 16 years and (4) university/advanced level (A-level)—A-level examinations taken around the age of 18 years.

#### Outcome measures

Survey respondents were asked the following questions: (1) ‘Has anyone in this household attempted suicide?’; and (2) ‘Has anyone died as a result of an attempted suicide?’ While we did not measure suicidal intent, the wording of the questions used communicates at least some intent. If an attempt was reported, respondents were asked when this occurred: last year, or over a year ago for any household member (past and present). The household member(s) who attempted suicide were recorded when possible, which enabled us to identify the individual household member who attempted suicide in 93% of cases. We only include suicide attempts in this analysis because demographic survey data were not collected for deceased individuals. In order to check for potential biases introduced as a consequence of failing to match cases within households, we did a sensitivity analysis.

### Statistical analysis

For this study, we only included individuals who were ≥10 years old because there were no reported suicide attempts under this age. The analysis was restricted to suicide attempts reported to have occurred in the past year to ensure the assumption that SEP and other factors measured at the time of the baseline survey were relevant at the time of the event. We conducted a complete case analysis, excluding individuals with missing data (see online supplementary table S1). In order to investigate whether the exclusion of those with missing data biased our results, we compared the characteristics of cases with complete versus incomplete data. In the regression models, for categorical variables we used the highest SEP as the reference category, except for occupation, where we used farmers as the reference category, as this was the largest group at the household level.

Our data are clustered (people within households, median household size=3 people); therefore, we used random-effects logistic regression models to investigate associations of the various SEP measures with suicide attempts; such models account for clustering effects. All models are adjusted for age and gender; crude associations are available in the online supplementary material (table 2). We used households as the unit of clustering. Given that many of the SEP factors are highly correlated, we investigate the association of each SEP factor separately. We also tested for age and gender differences using tests of statistical interaction; for these analyses, age was categorised into two (roughly equal size) groups.

**Table 2:** Age-adjusted, sex-adjusted and sex-stratified associations of SEP measures with a respondent report of attempted suicide in the past year

	Overall (age-adjusted/sex-adjusted)		Sex-stratified OR* (95% CI)		
	OR (95% CI)	p Value	Female	Male	p value for interaction†
*Household measures*					
Asset score					
High	1	<0.001‡	1	1	0.22
Middle	1.85 (1.49 to 2.29)		1.75 (1.33 to 2.34)	1.96 (1.41 to 2.72)	
Low	3.21 (2.36 to 4.37)		2.44 (1.56 to 3.81)	4.30 (2.80 to 6.57)	
Highest occupation in household§					
Government worker/graduate foreign employed	0.62 (0.41 to 0.93)	<0.001§	0.51 (0.28 to 0.94)	0.76 (0.44 to 1.32)	–
Farmer	1		1	1	
Security forces	1.03 (0.72 to 1.47)		1.60 (1.06 to 2.42)	0.40 (0.18 to 0.87)	
Businessmen	0.70 (0.36 to 1.33)		0.90 (0.41 to 1.97)	0.46 (0.14 to 1.44)	
Self-employed	1.28 (0.94 to 1.74)		1.13 (0.73 to 1.74)	1.46 (0.95 to 2.24)	
Non-graduate foreign employed	1.08 (0.59 to 1.96)		1.08 (0.49 to 2.36)	1.11 (0.45 to 2.74)	
Salaried employee	1.11 (0.75 to 1.65)		1.38 (0.85 to 2.23)	0.78 (0.39 to 1.55)	
Daily wage labourer	2.25 (1.61 to 3.16)		1.79 (1.09 to 2.94)	2.76 (1.76 to 4.31)	
Unemployed/retired	1.41 (0.65 to 3.06)		1.15 (0.36 to 3.70)	1.81 (0.65 to 5.00)	
Other¶	2.42 (0.97 to 6.04)		3.98 (1.55 to 10.23)	–	
Young female head of household (≤40 years)	1.60 (0.86 to 2.97)	0.14	2.30 (1.24 to 4.23)	–	–
Household with non-graduate foreign employed	0.87 (0.62 to 1.23)	0.43	0.88 (0.56 to 1.39)	0.86 (0.51 to 1.47)	0.99
*Individual measures*					
Individual occupation§					
Government worker/graduate foreign employed	0.25 (0.09 to 0.69)	<0.001‡	–	0.49 (0.18 to 1.37)	–
Farmer	1		1	1	
Security forces	0.23 (0.10 to 0.51)		–	0.26 (0.12 to 0.58)	
Businessmen	0.25 (0.06 to 1.02)		–	0.36 (0.09 to 1.48)	
Self-employed	1.18 (0.76 to 1.82)		1.78 (0.79 to 4.02)	1.08 (0.64 to 1.81)	
Non-graduate foreign employed	0.19 (0.06 to 0.63)		0.35 (0.10 to 1.28)	–	
Salaried employee	0.85 (0.57 to 1.28)		1.20 (0.58 to 2.49)	0.75 (0.44 to 1.29)	
Daily wage labourer	1.82 (1.22 to 2.72)		2.17 (0.83 to 5.65)	1.90 (1.22 to 2.95)	
Unemployed/retired	1.42 (0.95 to 2.12)		1.60 (0.77 to 3.29)	1.76 (1.05 to 2.93)	
House worker/other	0.78 (0.53 to 1.14)		1.04 (0.55 to 1.98)	0.57 (0.08 to 4.13)	
Student	0.23 (0.15 to 0.36)		0.35 (0.16 to 0.73)	0.14 (0.06 to 0.32)	
Individual education					
University/A-level	1	<0.001‡	1	1	<0.001
O-level	1.74 (1.29 to 2.35)		1.70 (1.18 to 2.45)	1.96 (1.16 to 3.30)	
Primary	2.72 (1.81 to 4.08)		2.57 (1.47 to 4.47)	3.11 (1.66 to 5.84)	
Not attended	4.63 (2.54 to 8.41)		3.10 (1.18 to 8.16)	8.35 (3.72 to 18.71)	

No p value for interaction presented when it was not possible to calculate interaction parameters due to limited data points.

*Estimated ORs adjusted for age.

†This is an interaction test with sex and does not adjust for age in the models. Cells with no data (–) are due to no strata-specific observations being present.

‡Likelihood ratio test comparing model with and without an SEP variable. The p value indicates whether there is any evidence that the suicide attempt rate varies across the groups.

§Presented according to ranking.

¶Made up of house workers, students and other occupations.

SEP, socioeconomic position.

The data set does not include information on psychiatric illness, a well-documented risk factor for suicidal behaviour. However, had measures of psychiatric morbidity been available, we would not have included them in our final model because psychiatric morbidity is likely to lie on the causal pathway. It could be, for example, that an individual's poor mental health contributed to their lower SEP (ie, social selection), or that an individual with a lower SEP is more vulnerable to developing a psychiatric disorder (ie, social causation). Therefore, models including psychiatric illness concurrently with SEP are likely to reduce the strength of the associations which is probably an overadjustment.

Some (34%) household members were not present in the household throughout the year (absent 1–11 months (30%) or <30 days (4%)). A slightly higher proportion of individuals from households with a low asset score were living away from home compared with individuals in households with a middle/high asset score. It is possible that the respondent may not have known about suicide attempt(s) made by the household members (as they occurred elsewhere), and that this could have influenced any associations. To investigate this, we carried out a sensitivity analysis restricting the study sample to those individuals living at home throughout the preceding 12 months. We also investigated the impact of respondent type on the association between lower levels of education (the SEP measure with the strongest association) and attempted suicide in the past year. We did this because certain respondents may be more or less likely to know about a past suicide attempt. For example, households in which the head of the household was the respondent may be more likely to report more accurately on past suicide attempts than a household without the head of household as a respondent (eg, if a young adult offspring had not been informed of one of their parents' suicide attempts).

## Results

### Study characteristics

**Figure 1:**
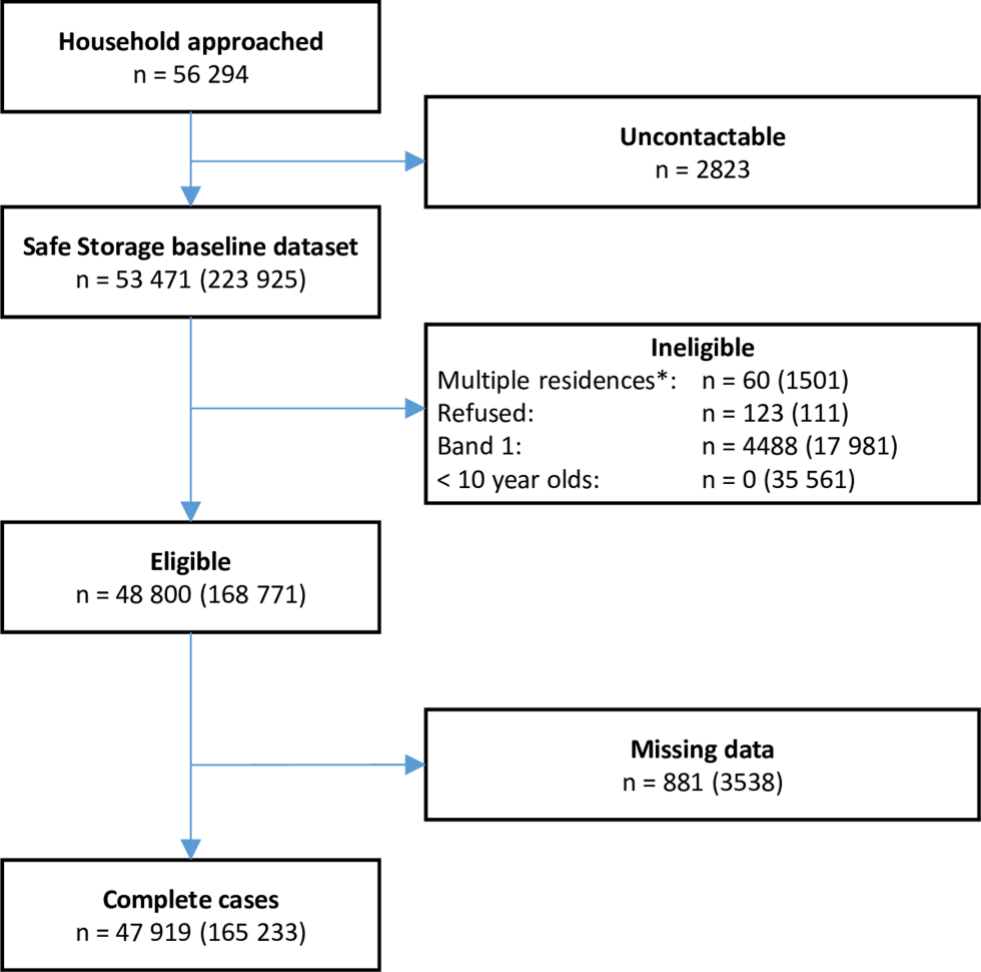
Flow chart of households/individuals included in the analysis. *Households/individuals who live in more than one place. n=households (individuals).

The Safe Storage trial surveyed 53 471 households (223 925 individuals) with a 95% response rate; 47 919 households (165 233 individuals) with complete data were eligible for inclusion in this analysis (figure 1). A comparison of eligible participants (n=168 771) with complete (n=165 233 (98%)) versus incomplete data (n=3538 (2%)) suggests that incomplete cases were somewhat more likely to be from households with a non-graduate foreign employed individual (12.4% vs 10.2%). The distribution of SEP is presented in table 1. The median household size was 3 members (data not shown), with the majority of individuals (65%) living in households with a high asset score. Only 1.5% of individuals lived in a household with a young (≤40 years) female head of household.

There were 398 (175 male; 223 female) individuals with a respondent-reported suicide attempt in the 12 months prior to data collection. These gave rates of 239 per 100 000 in ≥10 years old; 266 per 100 000 in women and 215 per 100 000 in men. Young women (10–25 years of age) had the highest incidence (530 per 100 000), whereas older women (56+ years) had the lowest rate (14 per 100 000).

### Associations of SEP with attempted suicide

Households with fewer assets were more likely to report an attempted suicide (p value for trend <0.001; table 2). In age and sex adjusted models, households with daily wage labourers (OR 2.25) as the highest occupation of the household were more likely to include individuals who had a respondent-reported attempted suicide (table 2). There were five cases of attempted suicide in households with an individual with an ‘other’ occupation as the highest occupation of the household; all five cases occurred in households with a female house worker as the highest occupation of the household. In terms of each individual's occupation, being a daily wage labourer showed a higher risk of a respondent-reported attempted suicide compared with farmers. Most other occupations had a reduced risk of attempted suicide compared with farmers; government workers/graduate foreign employed, security forces, businessmen and students (OR≤0.25) had the lowest risk. Lower levels of education increased the risk of attempted suicide (p value for trend <0.001). There was no statistical evidence of an increased risk in individuals from a household with a non-graduate foreign employed member or a young (≤40 years) female head of household.

### Age/gender differences in associations

There was strong statistical evidence that associations of education with respondent-reported suicide attempts differed in men and women (p value for interaction ≤0.001; table 2). The association of lower levels of education was greater in men than women, with those having not attended school showing the most marked difference (nearly threefold) between men and women.

It was only possible to calculate interaction parameters for age with asset score, households with non-graduate foreign employed individuals and education. There was no statistical evidence of a modifying effect of age (p value for interaction ranged from 0.13 to 0.79).

### Sensitivity analysis

We conducted four sensitivity analyses. Effect estimates from analysis of all households with a suicide attempt, regardless of whether they were matched to individuals, indicated similar results to our main analysis (see online supplementary results—table S3). When we excluded individuals not present in the household throughout the year, the associations of respondent-reported suicide attempts with SEP tended to be attenuated but did not alter our conclusions (see online supplementary results—table S4). Adjusting for respondent type had little impact on the effect estimates of the main analysis (see online supplementary results—table S5). Lastly, we investigated whether changing the age cut-off to define young female heads of household had an impact on our findings. Individuals in households with a very young female head of household (≤30 years, n=320) had a 4.8 (95% CI 1.3 to 13.6) greater risk of an attempted suicide, compared with 1.6 (95% CI 0.7 to 3.0) with an age cut-off of 40 years or younger. An older age cut-off (≤50 years, n=8234) showed a similar magnitude of effect (OR 1.4, 95% CI 1.0 to 2.1) to the 40 years age cut-off. An additional post hoc analysis comparing the risk of the young female heads of household themselves to that of other women of a similar age showed statistical evidence that being a young female head of household (regardless of the age cut-off used) increased the risk of attempted suicide by 2–6 times in these women.

## Discussion

### Main findings

The findings of this cross-sectional study in rural Sri Lanka suggest that poorer SEP, as measured by asset score, occupation and lower educational attainment, increases the risk of respondent-reported attempted suicide. The strongest association (fourfold increased risk) was with not having attended school. This association was considerably stronger in men than women. Individuals engaging in daily wage work (ie, insecure low-income jobs, regardless of gender) had the highest associated risk of attempted suicide, as did being from a household with a daily wage worker as the highest occupation.

### Comparison to other studies

Our findings are consistent with other similar studies in this region which have shown a higher risk of suicide/attempted suicide with lower levels of assets^24–26^ and education.^27–31^ Our study design, however, differs from previous research as we include a general population sample (no age/gender restrictions) and do not concurrently adjust for several measures of SEP as such adjustment may lead to underestimation of effects due to collinearity between measures.^14^ The strength of the association with lower assets was similar to findings from case–control and cross-sectional studies,^25^ ^26^ but twice as large as an estimate from a cohort study conducted in India.^32^ The strength of the association of lower levels of education with attempted suicide risk was in the middle of the range of estimates from previous studies (OR 2.7–5.7).^29–31^ The increased risk of attempted suicide among people with lower levels of education is also consistent with findings from South-East Asian HIC.^33^ Relatively few studies have investigated the association of occupation categories with suicide/attempted suicide in general population samples in LMIC in South and South-East Asia.^26^ ^32^ ^34–36^ Findings from these studies are mixed, but two cohort studies indicated that the risk of suicide is higher in manual compared with non-manual occupations (RR 1.2–1.4);^32^ ^36^ this is similar to evidence from South-East Asian HIC.^37^ The finding from the present study that the risk of attempted suicide was higher in farmers compared with non-manual occupations is consistent with previous evidence.^32^ ^36^

Previous studies in primarily western HIC have shown that the association of income, education level, occupation and employment status with suicide/attempted suicide may differ in men and women.^11^ ^17^ ^18^ ^38^ We found evidence that the effect of education level was modified by sex, with a stronger association in men. In Sri Lanka, there are very clear gender roles. Men are traditionally the main earners and have responsibilities for economic provision for the family. Men who struggle to meet these expectations (ie, those with a lower SEP) may experience significant psychological distress which might put them at a higher risk of attempted suicide compared with women with a similar SEP.

### Possible mechanisms

The exact mechanisms through which each of the indicators of SEP included in this study increases the risk of attempted suicide need to be explored in further detail. It has been suggested that greater levels of economic adversity lead to higher levels of anxiety, hopelessness and entrapment,^39–41^ and therefore to a suicide attempt. Strategies designed to alleviate some of the associated distress in individuals with lower levels of SEP may reduce the number of suicide attempts. Evidence suggests that farmers in this region (India) are shouldering large amounts of debt sourced from non-institutional credit sources (ie, moneylenders) which charge high interest rates.^42^ Potentially providing alternative regulated credit sources may aid suicide prevention efforts in this region.

The role of alcohol misuse and domestic violence are other important factors to consider in the association between SEP and suicidal behaviour. Individuals from a lower SEP (particularly daily wage labourers) in Sri Lanka are more likely to drink illicit alcohol, which is often associated with problem alcohol use and intimate partner violence,^43^ ^44^ and in turn suicidal behaviour in the individual and other household members.^45–47^ Lower SEP has also been linked to higher levels of intimate partner violence,^48^ which in turn has been shown to be associated with increased suicidality in women.^49^ Both these factors are difficult targets for intervention, but nevertheless their importance in suicidal behaviour needs to be recognised.

### Strengths and limitations

To the best of our knowledge, this is the first large representative study in a general population sample investigating the associations of SEP with attempted suicide in South and South-East Asia. The study achieved a very high response rate (95%) and included data on a range of SEP indicators.

There are, however, several limitations to this study which need to be considered. First, individuals from higher SEP households may be less likely to report an attempted suicide because of the stigma associated with suicidal behaviour. This may have resulted in an overestimation of the association of SEP with attempted suicide. Second, for pragmatic reasons we interviewed the adult members of the household who were home when we visited. It may be possible that certain respondents may be more or less likely to be knowledgeable/willing to report past suicide attempts, which may have impacted on our findings. Our sensitivity analysis did not suggest that our results were unduly affected by respondent type (see online supplementary results—table S5). Third, our data collectors were primarily young adults (18–24 years old) and the interview was conducted face to face. It is possible that the age and gender of the data collector might have influenced the willingness of the respondent to report a suicide attempt. In order to limit the impact of this, data collectors underwent intensive initial and ongoing training, and were regularly shadowed by supervisors. This was to ensure that the interviews were conducted in a way to establish rapport with household members and thereby encourage respondents to feel comfortable to answer the sensitive questions of the survey. Fourth, since this is a cross-sectional study, we are unable to determine the temporal relationship of the association of attempted suicide with SEP; it could be that the associations we have observed are due to reverse causality, that is, the suicide attempt resulted in disability which led to a loss of income/job. This may be an alternative explanation for transient SEP measures such as occupation and gender of head of household, but is unlikely to be the case for SEP indicators which are formed over time (eg, education/ assets). Lastly, we may have introduced a bias into our study by excluding the small number of cases (7%) which we were unable to link back to demographic details. Our sensitivity analysis, however, indicated that the exclusion of these cases would not have impacted on our overall conclusions.

## Conclusions

This large representative study in rural Sri Lanka indicates that lower levels of SEP (particularly lower levels of assets; education and having an unstable occupation) are associated with an increased risk of a suicide attempt in the past year. These findings are generalisable to other rural Asian populations and are similar to findings from HIC. The strongest associations with a suicide attempt were with lower levels of education. Our findings suggest that suicide prevention strategies targeted at particular groups of individuals (eg, very young female-headed households or men with no education) may be important. It is important to also acknowledge that only 1% of all suicide attempts occurred in very young female-headed households, and 3% in men with no education. The findings from this study, which relies on respondent report for the measure of outcome, and may be subject to reverse causality due to the cross-sectional design, should be replicated in a prospective cohort study with objective measurement of outcome.
