# Supplementary material for: Is socioeconomic position associated with risk of attempted suicide in rural Sri Lanka? A cross-sectional study of 165 000 individuals
Source: BMJ Open. 2017 Mar 22;7(3):e014006. doi: 10.1136/bmjopen-2016-014006 (PMC5372106; doi:10.1136/bmjopen-2016-014006)
Supplement: supplementary data [file bmjopen-2016-014006supp.pdf]

Supplementary materials

Supplementary methods

Data Collectors Guide

The data collectors were trained in Sinhala using the following questions

Introduction and consent:

- We are from Peradeniya University/Medical faculty/ research center
- We work with the provisional directors office in Anuradhapura
- We are conducting a survey to understand health problems in the area
- We have notified the GN and other officials in the area about this survey
- The data we will collect will include some basic information (similar to what is collected in the census), information about farming patterns and other related health information
- The study will be important in the future in order to help plan health services in your area
- We will require 15 minutes of your time
- If you cannot or don't wish to answer any of the questions that is not a problem and you can stop at any time
- Are you happy to take part in this survey?

Locating Household

- Do you remember this household's household number?
- Can you tell me the full name, with the family name, of the head of household?

Village Name

- What is this village's name?
- If you go to hospital what do you give as the village name?

Postal Address

- What is the address of this house if receiving mail (along with the house number)

Pesticide Use

Introduction

- Do you do any farming?
- Did you farm in the last maha/yala or this current season?
- Do you cultivate in your home garden?

Pests

- For the things you grow do you get trouble from worms/pests/weeds?

Pesticide use (farm land)

- What did you do when you experienced threats from insects?
- What do you do to control weeds?

Pesticides use (home garden)

- For the crop you grow in your home garden, if you have problems with worms/pests/weeds what do you do to clear them?

Timing

- In the last maha/yala did you use pesticides?
- How many months since you last used pesticides in your home garden?

Pesticide storage

- When you buy the pesticides to spray either in the field or in your home garden, where do you keep them?
- How about unopened bottles?
- How about the leftovers after you have sprayed the field, where do you keep them?
- The pesticides to be used in the garden, where do you keep these?
- As a habit where do you usually keep the pesticide bottles?
    - Inside the house
    - In the garden
    - In a shed in the garden
    - In the paddy field
    - In a 'chena' field or an abandoned land?

Locking

- Do you lock in pesticides when it's at home?
- Can you lock this room or box/compartment?

- Is it locked at all time?

Relocation

- Does anyone from this household go away to another area and stay overnight to do farming?
- For how many days did you relocate in last maha/yala season?
- Who relocates from the household?
- Where do you relocate to? (closest village)

Individual details *(these details related to both respondent and other household members)*

- Now I am going to take details for the people in the household – how many people have national identity cards (NICs) made?
- If it's no trouble can I get the NICs to have a look at the NIC number?
- All together how many people live in this household?
- Are there any additional relatives or children that live here?

Head of household

- Who does the work/acts as head of household?

Family Name (where no NIC)

- Can you give me the full name, including their family name?

Name with initials

- How is your/their name with initials?
- If you got to hospital how do you give you name normally?

Other Name

- What do the neighbors/villagers call you?
- In addition to this name are there any other names that are used?

Where no NIC is available

- Is there anywhere you have written down the NIC number? For example a photocopy?

Birthday/Age

- When is your/their date of birth?
- How old are you? (*used to confirm details on the NIC are accurate)*

Frequency

- Do you/they spend most of your time at home?
- From last year *July* till now, did you go and stay away from the home to visit a relative, to stay in hospital, for work?
- If you/they do stay, how long do they stay away for?

Education

- How far have you/they gone through school?
- What grade/class did you/they get to in school?
- *If the respondent reports that the household member did not attend school and the individual is elderly, then ask*: Can this person read/write?

Occupation

- At the moment what are you/they doing for your/their main job?
- In addition to housework do you/they do any other work?

Additional people

- In addition to those people you have told me about, are there any other people, relatives, workers who are staying here?

Vehicle ownership

- If you had to go in an emergency to the hospital, in the middle of the night, how would you get there?
- How do you find vehicles for business or field work?
- Does this house have any bicycles/ motorbikes/ tractors/ three wheelers/ cars/ vans/ lorries? (*This question should be asked to know if they have any vehicles not mentioned before. For example: if it was mentioned that they use a three wheeler to commute to the hospital, you can ask if they have access to a bicycle or a four wheeler.)*

Alcohol

Introduction

- So far I have asked details about the family, but now I will ask questions related to health. We are doing a health survey, the reason why we took individual details is so that we can match this with the information we are collecting in hospital. Like our team there is another team working in hospitals collecting details. We hope that we will be able to match the two details to understand what health problems are affecting this village. Therefore the accurate details we collect from the

village are very valuable. In addition we have two other health problems to ask. It is only with your cooperation that we can identify and help relieve a health problem affecting this area. These question may be relevant to you or irrelevant, but we have to ask each question to find out whether they are relevant. We only take household information from the household and therefore we have to ask both relevant and irrelevant questions in order to get an accurate answers.

Consumption

- Does anyone in this house consume alcohol?
- Does anyone use beer during functions or occasionally at home

Problem

- Does anyone in this household perceive this use as a problem/trouble?

Suicide and attempted suicide

Introduction

- Has anyone in this household, it can be recently or from a long time ago ever had an allergy or adverse reaction to a medicine or food?
- Has anyone in this household, it can be recently or from a long time ago, perhaps even from before you were born or started living here, ever accidently eaten a seed or taken a poison? How about during your childhood?
- In life there are a lot of problems, some people can face these problems, some people cannot. In these situations some people may not be able to deal with the problem by talking to someone but some may decide to make an attempt on your life.

Question

- Has anyone in this household attempted suicide?
- Maybe not recently but any time where your memory can recollect?
- Has anyone died as a result of attempted suicide?
- Approximately how long ago was this?
- What is his/ her full name?
- Was that a first attempt or has he/ she attempted before?
- What/ how did he/ she use to for this attempt?

Details on composite score

We used household construction as a measure of accumulated wealth and used a similar method for defining household construction as used by the Sri Lankan census department. This method classifies households based on the principal materials used in the construction of the walls, roof and floor. Data collectors would assess the materials used and classify these according to whether materials used were durable (e.g. bricks, cement) or non-durable (e.g. mud, straw). If the materials used for the walls, roof and floor of the main room of a household were mainly: durable, these were classified as having a solid construction (high); non-durable, these were classified as improvised (low); mixture of both durable and non-durable, these were semi-permanent (middle). In order to ensure consistency of assessing household construction we held regular training events and assessments to ensure inter-rater reliability. We also carried out repeatability checks in a random selection (3%) of households in 6 bands. The inter-rater reliability was good (Kappa = 0.67, 95% CI 0.59 to 0.74).

Data on motorised vehicle ownership was obtained via direct questioning of household members, whereas household construction was recorded by the data collectors based on construction materials of the house

Details on occupation ranking exercise

In order to generate a rank of occupations in terms of social prestige, we conducted a ranking exercise with villagers. The ranking was done with 20 different occupations which were mapped onto the occupation categories outlined in table 1. We did not include house-worker, student or “other” (e.g. clergy, commission based jobs, volunteers and politicians) as occupation categories in the ranking exercise as these occupations are difficult to rank. Therefore we combined those into a single category. Table 1 (main manuscript) shows the occupation categories according to the ranking developed; government workers and graduate foreign employed individuals were at the top of the ranking followed by farmers, with unemployed/retired individuals at the bottom of the hierarchy. There were no households where the highest ranking occupation was an individual in the “other” category. In households where a house-worker/student was the highest occupation, 99% were house-workers with only 1% students.

Supplementary results

Supplementary table 1 – Number of participants with missing data for variable included in the analysis

	Missing n (%)
**Household measures**	
Asset score	307 (0.18)
Highest occupation in household	1 (0)
Young female head of household (≤40 years)	31 (0.02)
Household with non-graduate foreign employed	0 (0)
**Individual measures**	
Individual occupation	99 (0.06)
Individual education	340 (0.20)

Supplementary table 2 – Crude associations of SEP measures with a self-report of attempted suicide in the last year

	OR	95% CI
**Household measures**		
Asset score		
High	1	
Middle	1.78	1.43, 2.21
Low	3.16	2.31, 4.32
Highest occupation in household		
Government worker/Graduate foreign employed	0.60	0.40, 0.90
Farmer	1	
Security forces	1.10	0.77, 1.56
Businessmen	0.71	0.37, 1.35
Self-employed	1.32	0.97, 1.80
Non-graduate Foreign employed	1.10	0.61, 2.00
Salaried employee	1.16	0.78, 1.72
Daily Wage labourer	1.89	1.01, 3.53
Unemployed/retired	0.78	0.19, 3.20
House-worker/Student/Other	2.06	1.47, 2.90
Young female head of household	1.92	1.03, 3.55
Household with non-graduate foreign employed	0.88	0.62, 1.24
**Individual measures**		
Individual occupation		
Government worker/Graduate foreign employed	0.33	0.12, 0.90
Farmer	1	
Security forces	0.42	0.19, 0.93
Businessmen	0.30	0.07, 1.24
Self-employed	1.56	1.01, 2.40
Non-graduate Foreign employed	0.34	0.11, 1.11
Salaried employee	1.91	1.30, 2.79
Daily Wage labourer	2.46	1.65, 3.66
Unemployed/retired	1.79	1.24, 2.57
House-worker/Student/Other	1.11	0.81, 1.52
Individual education		
University/A-level	1.00	
O-Level	1.57	1.16, 2.12
Primary	1.21	0.82, 1.80
Not attended	1.70	0.95, 3.05

Supplementary table 3 - Household level analysis of attempted suicide reports in the last year (clustering at area level accounted for)

	Matched cases n=402 n(%)	Unmatched cases* n=30 n(%)	OR**	95% CI
Household asset score				
High	192 (47.8)	18 (60.0)	1	
Middle	156 (38.8)	10 (33.3)	1.54	1.25,1.89
Low	54 (13.4)	2 (6.7)	2.33	1.72,3.13
Young female headed household	11 (2.7)	1 (3.0)	1.58	0.88,2.82
Households with non-graduate foreign employed	37 (9.2)	3 (10.0)	1.12	0.81,1.56
Highest occupation in a household				
Government worker/Graduate foreign employed	27 (6.7)	1 (3.3)	0.61	0.40, 0.90
Farmer	167 (41.5)	19 (63.3)	1	
Security forces	38 (9.5)	2 (6.7)	0.94	0.66, 1.32
Businessmen	10 (2.5)	0 (0)	0.57	0.30, 1.08
Self-employed	56 (13.9)	3 (10.0)	1.10	0.81, 1.48
Non-graduate Foreign employed	12 (3.0)	0 (0)	1.01	0.56, 1.82
Salaried employee	31 (7.7)	1 (3.3)	0.97	0.67, 1.42
Daily Wage labourer	11 (2.7)	1 (3.3)	1.23	0.68, 2.22
Unemployed/retired	2 (0.5)	0 (0)	0.31	0.08, 1.25
"Other"	48 (11.9)	3 (10.0)	1.50	1.09, 2.04

**Any household with at least one unmatched case*
*** Clustering at area level accounted for in model. Analysis of household level factors regardless of whether an individual match was made to the reported suicide attempt (i.e. comparison of 432 households with attempts to non-attempting households)*

Supplementary table 4 – Comparison of associations of SEP with suicide attempts in the last year stratified by frequency at home

	OR (95% CI)	
	Unrestricted*	Restricted**
<u>Household measures</u>		
Asset score		
High	1	1
Middle	1.85 (1.49, 2.29)	2.07 (1.57, 2.73)
Low	3.21 (2.36, 4.37)	2.76 (1.77, 4.31)
Highest occupation in household		
Government worker/Graduate foreign employed	0.62 (0.41, 0.93)	0.59 (0.35, 1.01)
Farmer	1	1
Security forces	1.03 (0.72, 1.47)	0.90 (0.54, 1.52)
Businessmen	0.70 (0.36, 1.33)	0.68 (0.32, 1.49)
Self-employed	1.28 (0.94, 1.74)	1.03 (0.68, 1.56)
Non-graduate Foreign employed	1.08 (0.59, 1.96)	1.44 (0.66, 3.14)
Salaried employee	1.11 (0.75, 1.65)	1.02 (0.60, 1.74)
Daily Wage labourer	2.25 (1.61, 3.16)	1.87 (1.20, 2.90)
Unemployed/retired	1.41 (0.65, 3.06)	1.51 (0.60, 3.79)
"Other"	2.42 (0.97, 6.04)	3.30 (1.67, 9.32)
Young female head of household (≤40 years)	1.54 (0.84, 2.82)	1.55 (0.67, 3.56)
Household with non-graduate foreign employed	1.60 (0.86, 2.97)	1.05 (0.64, 1.71)
<u>Individual measures</u>		
Individual occupation		
Government worker/Graduate foreign employed	0.25 (0.09, 0.69)	0.51 (0.18, 1.45)
Farmer	1	1
Security forces	0.23 (0.10, 0.51)	-
Businessmen	0.25 (0.06, 1.02)	0.18 (0.02, 1.33)
Self-employed	1.18 (0.76, 1.82)	1.19 (0.68, 2.06)
Non-graduate Foreign employed	0.19 (0.06, 0.63)	-
Salaried employee	0.85 (0.57, 1.28)	1.05 (0.60, 1.83)
Daily Wage labourer	1.82 (1.22, 2.72)	1.51 (0.86, 2.64)
Unemployed/retired	1.42 (0.95, 2.12)	1.52 (0.92, 2.53)
House-worker/Other	0.78 (0.53, 1.14)	0.76 (0.47, 1.21)
Student	0.23 (0.15, 0.36)	0.26 (0.15, 0.44)
Individual education		
University/A-level	1	1
O-Level	1.74 (1.29, 2.35)	1.49 (1.01, 2.19)
Primary	2.72 (1.81, 4.08)	2.15 (1.28, 3.62)
Not attended	4.63 (2.54, 8.41)	2.75 (1.18, 6.43)

**Include all individuals regardless of their time at home. **Restricted to those individuals who are reported to be home throughout the preceding 12 month*

Supplementary table 5 - Associations of education and suicide attempt risk in the last year adjusted for respondent type

	OR (95% CI)				
	Model 1	Model 2	Model 3	Model 4	Model 5
Individual education					
University/ A-level	1	1	1	1	1
O-Level	1.74 (1.29, 2.35)	1.74 (1.29, 2.35)	1.77 (1.31, 2.4)	1.74 (1.29, 2.35)	1.74 (1.29, 2.35)
Primary	2.72 (1.81, 4.08)	2.75 (1.83, 4.14)	2.8 (1.86, 4.21)	2.75 (1.83, 4.15)	2.75 (1.83, 4.14)
Not attended	4.63 (2.54, 8.41)	4.65 (2.55, 8.49)	4.66 (2.55, 8.51)	4.67 (2.55, 8.52)	4.67 (2.56, 8.53)

Model 1 – Adjusted for age and sex
Model 2 – Adjusted for age, sex and whether the head of household was a respondent
Model 3 – Adjusted for age, sex and whether a child (<10 years) was present/responded during the survey
Model 4 – Adjusted for age, sex and whether there was a single or multiple respondent(s)
Model 5 – Adjusted for age, sex and the gender make-up of respondents (i.e. male only, female only or mixed
